# Syringocystadenoma Papilliferum in the External Ear Canal

**DOI:** 10.34172/aim.2022.101

**Published:** 2022-09-01

**Authors:** Sasa Jakovljevic, Nenad Arsovic, Novica Boricic, Darko Laketic, Zoran Dudvarski

**Affiliations:** ^1^Clinic of Otorhinolaryngology and Maxillofacial Surgery, University Clinical Center of Serbia, Belgrade, Serbia; ^2^Faculty of Medicine, University of Belgrade, Belgrade, Serbia; ^3^Institute of Pathology, Faculty of Medicine, University of Belgrade, Belgrade, Serbia; ^4^Institute of Anatomy, Faculty of Medicine, University of Belgrade, Belgrade, Serbia

**Keywords:** Syringocystadenoma papilliferum, Ceruminous gland, External auditory canal

## Abstract

Out of all benign tumors of the ceruminous glands, syringocystadenoma papilliferum is the rarest and represents only 2% of cases. It is an extremely rare benign tumor that originates from modified apocrine sweat glands. The aim of this paper was to present, according to our findings, the 18th case of syringocystadenoma papilliferum in the external auditory canal, with a detailed review of its clinical, radiological and histomorphological characteristics. A 59-year-old man reported to our clinic due to a 5×5 mm papillomatous growth at the entrance to the right external auditory canal. Histopathology indicated, after an excisional biopsy, that it was a syringocystadenoma papilliferum. The resection lines were free of tumor tissue, and the patient has no signs of tumor recurrence. Although rare, it should be considered as a differential diagnosis of lesions in this region. Complete excision is mandatory in order to avoid recurrence and potential malignant alteration.

## Introduction

 Ceruminous glands are modified apocrine sweat glands located inside the dermis of the external ear canal (EEC). According to the literature, tumors of these glands are rare and represent only 5-5.7% of all external ear tumors, while some authors suggest an even lower frequency.^1-4^ In 1894, Haugh was the first to describe a tumor of the EEC ceruminous gland and proposed the term *ceruminoma*.^5^ According to histological characteristics, benign tumors of these glands include ceruminous adenoma, ceruminous pleomorphic adenoma, and ceruminous syringocystadenoma papilliferum.^4^ Syringocystadenoma papilliferum is a benign, slow-growing hamartomatous neoplasm, which appears to be the rarest of these tumors, and accounts for only 2% of cases.^1^ It is usually present at birth or in early childhood. However, most of the cases described in the literature have been reported in adults.^6^ Given that it is extremely rare in the EEC, the literature is mainly limited to case reports. To date, fewer than 150 cases of ceruminous gland tumors developing in the EEC have been reported in the literature.^5^ Of this number, only 17 cases of syringocystadenoma papilliferum in the EEC have been described in the literature in English.^5^ This paper aimed to present, according to our findings, the 18th case of EEC syringocystadenoma papilliferum with a detailed review of its clinical, radiological and histomorphological characteristics.

## Case Report

**Figure 1:**
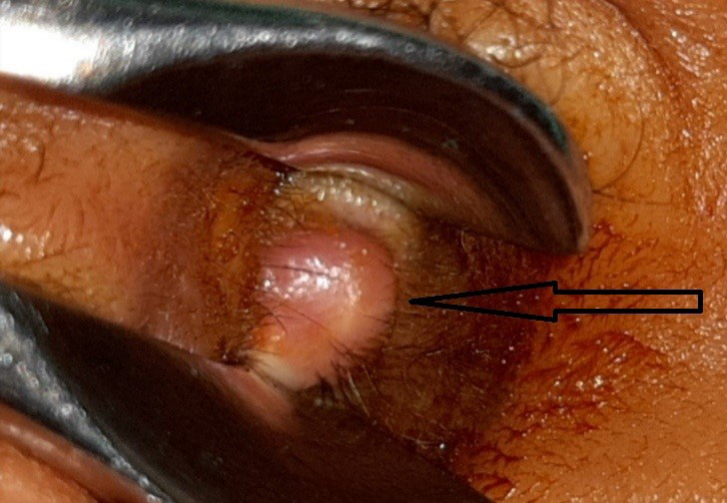
External Ear Canal of the Patient. The arrow shows the lesion before surgical removal

**Figure 2:**
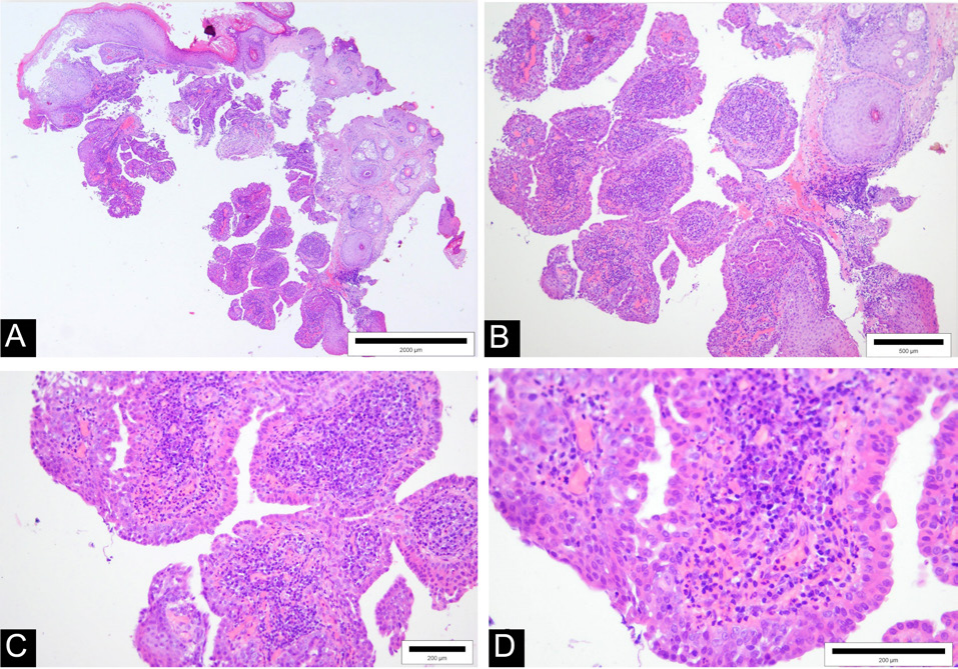
Histological Presentation of Syringocystadenoma Papilliferum (Hematoxylin-Eosin Staining). Double layer of cells and stroma rich in inflammatory cells. Microscopic magnification x4 (a), x10 (b), x20 (c), x40 (d)

**Figure 3:**
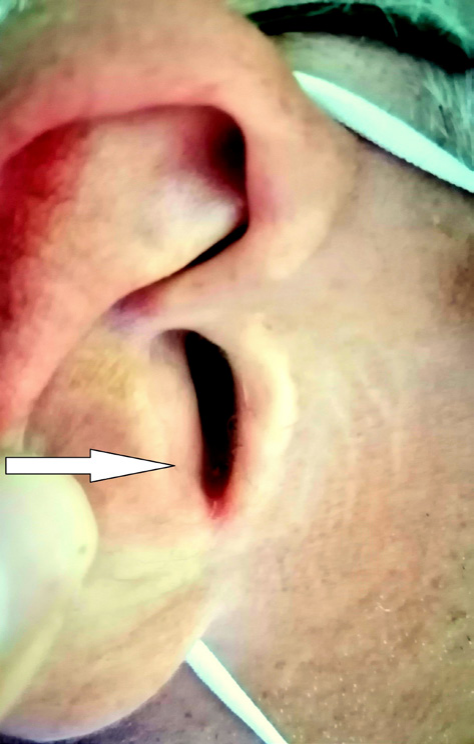
External Ear Canal, 14 Months After Complete Tumor Excision. The arrow indicates where the tumor was located before excision

 A 59-year-old man reported to our clinic due to the appearance of a growth at the entrance to the right external auditory canal and occasional mild bleeding from the right ear. Otoscopic examination found a papillomatous growth measuring 5 × 5 mm at the entrance to the EEC on the right side (Figure 1). The growth was reddish in color and not painful. The remaining findings in the EEC and on the eardrum were normal. Due to the size and benign appearance of the lesion, with clearly defined margins, no radiological examination was performed. Excision of the papillomatous growth with high-frequency radio waves under local anaesthesia was performed. The histopathological finding indicated that it was syringocystadenoma papilliferum (Figure 2A, B, C, D). Cystic intussusception was observed that opened onto the skin surface. Intussusception consisted of a double layer of cells – the inside layer composed of columnar cells and the outside layer formed of cuboid cells. Also, there was a stroma rich in inflammatory cells, mainly plasma cells. The resection lines were free of tumor tissue, and after 14 months of monitoring, the patient had no signs of tumor recurrence (Figure 3).

## Discussion

 Tumors arising from ceruminous glands of the EEC can cause a diagnostic dilemma due to different clinical and histological characteristics.^4^ They are relatively common in animals (cats and dogs), while they are rare in humans. A large number of authors have tried to classify the tumors of the ceruminous glands. Benign neoplasms of the ceruminous glands include ceruminous adenoma, ceruminous pleomorphic adenoma and syringocystadenoma papilliferum, while malignant neoplasms include adenocarcinoma, adenoid cystic carcinoma and mucoepidermoid carcinoma.^2^

 Syringocystadenoma papilliferum is an extremely rare benign tumor of the ceruminous glands with extensive papillary growth of epithelial elements in the dermis.^5^ In the head and neck area, it mainly occurs on the face and in the scalp area, and rarely in the EEC. It was originally named *“naevus syringadenomatosus papilliferum ”*, primarily due to its occurrence in the field of an already existing nevus, accounting for almost a third of all cases.^5,6^

 The etiology of syringocystadenoma papilliferum in the EEC is unknown and its histogenesis is controversial.^1,7^ Some authors have suggested that there is a possible connection between human papillomavirus infection and syringocystadenoma papilliferum.^5^ There is also evidence of connection with a change in the p16 tumor-suppressor gene (markers, IFNA and D9S171 have been identified).^2^ The literature also notes that this benign tumor is often connected with the existing nevi. In our case, there was no evidence of a nevus, but the tumorous change had a papillomatous appearance.

 This tumor may coexist with other benign lesions in the external auditory canal, such as chronic granulomatous otitis externa with myringitis, lipomatous and tubular apocrine adenoma.^1^ Although it is a benign tumor, malignant transformation into basal cell carcinoma has been also described, and syringocystadenocarcinoma papilliferum and ductal carcinoma have been noted.^6^ A number of other conditions should be considered in the differential diagnosis, such as cylindroma, papilloma, verrucous nevus, basaloid follicular hamartoma, pyogenic granuloma, neuroendocrine adenoma of the middle ear, parotid pleomorphic adenoma, meningioma and paraganglioma, malignant tumors of the ceruminous glands, squamous cell carcinoma and basal cell carcinoma, adenocarcinoma, and even certain diseases such as tuberculosis.^2-5,8^

 This benign tumor is initially mostly asymptomatic. Later, its growth can lead to the closure of the EEC and consequent symptoms such as hearing loss, otorrhagia, and mild to moderate otalgia.^1,2^ Otoscopic examination usually reveals a reddish or skin-colored lesion of various size and sometimes with superficial ulceration. Tumor size varies, but tumors larger than 2 cm have not been described in the literature, primarily because of the size of the EEC.^1,3^ The patient did not have hearing problems, did not have any pain, and referred to us because of the growth he noticed at the entrance to the EEC and occasional mild bleeding.

 Most authors prefer excisional over incisional biopsy. The sample obtained by incisional biopsy may be insufficient. Moreover, the presence of an intralesional cyst, which is of great help in diagnosis, may be lost by incisional biopsy. With an incisional biopsy, there is also a risk of not detecting malignancy and the development of hemorrhage or facial paralysis in the case of glomus tumors or schwannoma of the facial nerve.^2^ In our case, an excisional biopsy was performed.

 Ceruminous syringocystadenoma papilliferum can be accurately diagnosed on the basis of histological examination by staining with hematoxylin and eosin and without immunohistochemistry, as the tumor shows a unique papillary histological pattern. Histologically, it is a well-differentiated, cystic tumor with papillary proliferation of glandular tissue, histologically similar to that of normal ceruminous glands.^3,4^ A rich and dense plasmacytic infiltrate is also present in the fibrovascular stroma.^1,4,9^

 Syringocystadenoma papilliferum is mainly a histopathological diagnosis, and the role of radiology has not been well-established.^6^ In one of the rare reports on this topic, Kamakura et al have stated that magnetic resonance imaging may play a role in the diagnosis and differential diagnosis of syringocystadenoma papilliferum.^10^ On the other hand, CT of the temporal bone can help describe the tumor and rule out malignancy, which would be indicated by bone erosion or involvement of the middle ear.^6^ No radiological diagnosis was made in our patient, given that it was a minor tumor lesion of benign appearance.

 According to all the authors in the available literature, surgical excision is the gold standard in treatment. Treatment with CO_2_ laser has also been described, but mainly in pediatric patients.^6^ Radiotherapy and chemotherapy are not required in the treatment of these neoplasms.^1,2^ Periodic monitoring is recommended after complete excision as recurrences can occur in incompletely removed tumors. In the case of our patient, complete surgical excision was performed at the time of diagnosis, with resection lines having no tumor tissue. After 14 months of monitoring, the finding was normal.

 In conclusion, although syringocystadenoma papilliferum is a benign tumor of the EEC, it should be considered as a differential diagnosis of lesions in this region. The clinical presentation of the tumor is nonspecific, and an excisional biopsy with histopathological examination is necessary for a precise diagnosis. Immunohistochemical analysis is generally not necessary due to the characteristic histological appearance and the absence of infiltrative growth of the tumor. In order to avoid the occurrence of malignant transformation and recurrence, complete excision is required.
